# Computed tomography guided microwave ablation for the treatment of clinical T1a renal cell carcinoma: a comparison to robot-assisted laparoscopic partial nephrectomy

**DOI:** 10.1007/s00345-025-06037-x

**Published:** 2025-11-17

**Authors:** Rasmus D. Petersson, Thomas Bretlau, Munkith Abbas, Katrine S. Schou-Jensen, Frederik F. Thomsen

**Affiliations:** 1 Department of Urology, Zealand University Hospital, Sygehusvej 6, 4000 Roskilde, Denmark; 2 Department of Urology, Copenhagen University Hospital-Herlev and Gentofte Hospital, Herlev, Denmark; 3 Department of Radiology, Copenhagen University Hospital-Herlev and Gentofte Hospital, Herlev, Denmark

**Keywords:** Renal cell carcinoma, Microwave ablation, Partial nephrectomy, Robot-assisted, Computed tomography

## Abstract

**Purpose:**

To compare computed tomography guided microwave ablation (MW) and Robot-assisted laparoscopic partial nephrectomy (RAPN) for the treatment of T1a renal cell carcinoma (RCC).

**Method:**

Retrospective study including patients treated for clinical T1a RCC with either MW or RAPN.

**Results:**

In total, 71 patients were planned to MW, and 372 patients to RAPN. Patients undergoing MW were older, more comorbid, and had worse kidney function. The median follow-up was 2.4 years (95% CI 2.0, 3.4) and 3.7 years (95% CI 3.1, 4.0) for MW and RAPN, respectively. 6% of patients who underwent MW experienced a major (Clavien Dindo ≥ III) complication compared to 5% following RAPN. In uni- and multivariable logistic regression analyses, there was no significant difference in the risk of experiencing any or a major postoperative complication. The 5-year cumulative incidence of any recurrence was 10% for MW versus 4% for RAPN. The 5-year incidence of a distant recurrence was 0% for MW and 2% for RAPN, respectively. In univariable Cox regression analyses, patients treated with MW had a higher risk of experiencing any recurrence and a local recurrence compared to those who underwent RAPN, HR 4.24 (95% CI 1.26–14.4) for any recurrence and HR 6.95 (95% CI 1.83–26.4) for a local recurrence, respectively. There was no difference in the risk of a distant recurrence between treatment strategies.

**Conclusion:**

These results indicate that MW has an acceptable safety profile and oncological results, and as such is a feasible treatment option for patients with T1a RCC who are poor surgical candidates.

## Introduction

The preferred curative treatment modality for localized renal cell carcinoma (RCC) is partial nephrectomy (PN) when technically feasible, as it offers better preservation of renal function compared to radical nephrectomy without compromising oncological outcomes [1–3]. Robot-assisted laparoscopic partial nephrectomy (RAPN) is believed to be the safest surgical method for partial nephrectomy as it is associated with less morbidity, fewer complications and shorter hospital stay compared to open partial nephrectomy [4, 5].

However, surgical treatment carries inherent risks [6, 7], and not all patients are suitable for RAPN because of comorbidities or previous abdominal surgery [6–9]. Ablation therapy (AT) is an alternative treatment option for patients who are unfit for major surgery but still require treatment [10]. Recent data suggest that microwave ablation (MW) is more effective and equally safe as other AT modalities such as radiofrequency- or cryoablation [11].

The availability of multiple treatment options highlights the need for understanding the individual therapies and how they relate to each other, to guide treatment selection. Few studies have directly compared MW and RAPN [10], and most has a short follow-up period [12–14]. Thus, the objective of this study was to compare safety and oncological outcomes between MW and RAPN.

## Method

Retrospective study including all patients who underwent treatment for clinical T1a RCC with either computed tomography (CT)-guided MW or RAPN in 2017–2023 at the Department of Urology, Copenhagen University Hospital, Herlev and Gentofte Hospital, Herlev, Denmark. This study received ethical and legal approval from the Danish Society for Patient Safety according to Danish law (journal number: R-23070177). The requirement for individual informed consent was waived by the ethics committee due to the retrospective nature of the study, which involved the analysis of pre-existing clinical data.

MW procedures were performed using either the Emprint (Covidien, Boulder, USA) system or the TATO2 (Terumo, Tokyo, Japan) system. Patients undergoing MW had a biopsy verifying RCC, prior to treatment. Repeat CT scans were performed during treatment to confirm correct needle placement and a sufficient ablation area, 1–2 needles were used. If needed, hydrodissection was performed. The wattage ranged from 20 to 100 W, most often 40 W, and the ablation time was 5 to 30 min, with a median time of around 15 min. The cohort of patients undergoing RAPN has been described in a previous publication [15].

Patient treatment was determined following multidisciplinary team meetings and subsequent consultation between patient and treating physician. Although no formal criteria were available at our institution, MW was typically reserved for older, frailer patients and those with previous major abdominal surgery. The following data were recorded by electronic patient record review: preoperative data (age, gender, Charlson Comorbidity Index (CCI) [16], American Society of Anaesthesiologists classification (ASA) [17], body mass index (BMI), smoking status, estimated glomerular filtration rate mL/min/1.73 m² (eGFR), previous abdominal surgery, PADUA score [18] and tumor size), treatment type, complications within 90 days, eGFR one year after treatment, and oncological outcomes (recurrences, subsequent treatments).

Patients were followed postoperatively with systematic imaging, and clinical controls according to Danish National guidelines [19]. The follow-up schedule consisted of CT scans of the thorax and abdomen performed at 6 months, and then annually for the first five years, with subsequent scans at 7 and 9 years. Recurrences were identified after either routine imagining or imaging performed due to clinical symptoms suggestive of recurrence. Recurrence was confirmed by histological verification, which could be omitted in cases of unequivocal imaging. A recurrence in the ipsilateral kidney were considered a local recurrence, while all other recurrences were considered distant recurrences. No patient was lost to follow-up.

Descriptive statistics were used. Median follow-up was calculated with the reverse Kaplan–Meier method [20]. Odds ratios for postoperative complications were assessed with univariable logistic regression analyses: treatment modality (MW vs. RAPN), age (< 75 vs. ≥75), gender (female vs. male), CCI (0, 1, ≥ 2), ASA (2 vs. ≥3), BMI (normal: i.e. < 25, pre-obesity:25–30 vs. obese: >30), kidney function (CKD 1, CDK 2 vs. CKD ≥ 3), smoking status (smoker, former vs. never), tumor size (0-19.9, 20-29.9 vs. 30–40 mm). A multivariable analysis for any complication were subsequently performed including treatment modality (MW vs. RAPN) and CCI (0, 1, ≥ 2). There were too few major (CD ≥ III) complication events for any meaningful multivariable analyses. The difference between pretreatment eGFR and eGFR after one year was calculated. Cumulative incidence of recurrence was calculated with competing-risk analyses where death from non-RCC causes were treated as competing events. Hazard ratios (HR) for risk of recurrence for the different treatment modalities was estimated with univariable Cox regression analyses: treatment modality (MW vs. RAPN), tumor size (0–29.9 vs. 30–40 mm) and histology (clear cell vs. non clear cell). There were too few events to allow any meaningful multivariable analyses. Only patients treated for a primary RCC, verified by biopsy for MW or final pathology for RAPN, were included in the oncological analyses. Complication analyses included patients previously treated for RCC and those with benign pathology. All tests were two-sided, and the significance level was set to *p* < 0.05. Statistical analysis was performed with R version 4.3.3 (R Foundation for Statistical Computing, Vienna, Austria).

## Results

In total, 71 patients were planned to MW, and 372 patients to RAPN for a T1a RCC. Three MW-procedures were aborted peroperatively, because the tumor was too close to the renal pelvis or the colon, even after hydrodissection, leaving 68 patients for safety analyses. Seventeen patients underwent MW because of local recurrent RCC following partial nephrectomy, leaving 51 patients who underwent MW as the primary treatment of RCC and available for oncological analyses. All patients who underwent MW had ASA scores ≥ 2, we therefore excluded patients with ASA scores 1, leaving 308 patients who underwent RAPN Among these, 7 patients were converted to nephrectomy due to perioperative challenges, resulting in 301 patients for safety analyses. Benign histology was found in 35 patients undergoing RAPN, leaving 266 patients for oncological analyses.

The median follow-up was 2.4 years (95% CI 2.0, 3.4) and 3.7 years (95% CI 3.3, 4.1) for MW and RAPN, respectively. Baseline patient characteristics are presented in Table 1.

**Table 1:** Baseline data

Variable	Treatment received		*p* value^b^
	PN, *N* = 308	MW, *N* = 68	
	n (%)	n (%)	
Age^a^	64 (57, 71)	73 (68, 77)	< 0.001
Gender			0.054
Female	100 (32%)	14 (21%)	
Male	208 (68%)	54 (79%)	
CCI			< 0.001
0	167 (54%)	25 (37%)	
1	90 (29%)	17 (25%)	
≥2	51 (17%)	26 (38%)	
ASA			< 0.001
2	210 (68%)	30 (44%)	
≥3	98 (32%)	38 (56%)	
Body Mass index^a^	27.0 (24.0, 31.0)	27.0 (25.0, 31.0)	0.98
Preoperative eGFR^a^	88 (74, 90)	67 (58, 86)	< 0.001
Change in eGFR (1-year)	-3 (-11, 0)	-2 (-7, 0)	0.029
Previous abd. surgery			< 0.001
None	207 (67%)	30 (44%)	
Lap	39 (13%)	20 (29%)	
Open	62 (20%)	18 (26%)	
Smoking			0.054
Never	120 (39%)	16 (24%)	
Smoker	76 (25%)	20 (29%)	
Former smoker	112 (36%)	32 (47%)	
Tumor size (mm) ^a^	26 (20, 32)	25 (20, 30)	0.083
PADUA score	7(6, 8)	7(6, 8)	0.66

*PN* robot-assisted laparoscopic partial nephrectomy, *MW* microwave ablation, *CCI* Charlson comorbidity index, *ASA* American Society of Anaesthesiology score

^a^Median (IQR) or Frequency (%)

^b^Wilcoxon rank sum test; Pearson’s Chi-squared test

The cohorts were not comparable, with a higher median age, higher CCI, higher ASA score and worse kidney function among patients undergoing MW compared to patients who underwent RAPN. However, there was no significant difference in tumor size.

The hospital stay was shorter for patients undergoing MW compared to patients who underwent RAPN: 1.6 days (IQR 1–2) vs. 4.0 days (IQR 2–4), *p* < 0.001.

There were 4 (6%) intraoperative complications for patients undergoing MW: Three cases of uroplania, two managed with ureteric stent and one with conservative management. There was one pneumothorax, which was managed conservatively. Following RAPN 6 (1.6%) had an intraoperative complication; three spleen lesions, one diaphragm perforations, one kidney vein injury and one small bowel perforation. The complications were managed with either sutures and/or synthetic hemostatic patches.

Postoperative complications were recorded in 18 (26.5%) patients undergoing MW and 94 (31%) patients undergoing RAPN, Table 2.

**Table 2:** Overview of postoperative complications

	CD I	CD II	CD IIIa	CD IIIb	CD IVb	CD V
*Postoperative complications after microwave ablation*						
Infection	–	6 (8.8%)	1 (1.5%)	–	–	–
Other	2 (3.0%)	3 (4.4%)	–	–	–	–
Pain	3 (4.4%)	1 (1.5%)	–	–	–	–
Bleeding	–	–	–	1 (1.5%)	–	–
Pneumothorax	–	–	1 (1.5%)		–	–
Total	5 (7.3%)	10 (15%)	2 (3.0%)	1 (1.5%)	–	–
*Postoperative complications after partial nephrectomy*						
Infection	1 (0.3%)	47 (15%)	1 (0.3%)	1 (0.3%)	–	–
Bleeding	4 (1.3%)	5 (1.6%)	–	6 (1.9%)	–	–
Other	8 (2.6%)	4 (1.3%)	1 (0.3%)	–	–	–
Ileus	–	3 (1.0%)	–	–	–	–
Urinary retention	3 (1.0%)	–	–	–	–	–
Uroplania	–	–	–	3 (1.0%)	–	–
Kidney failure	–	–	–	–	–	2 (0.6%)
Bowel perforation	–	–	–	–	1 (0.3%)	–
DVT	–	1 (0.3%)	–	–	–	–
Hernia	–	–	–	1 (0.3%)	–	–
Pain	1 (0.3%)	–	–	–	–	–
TCI	–	1 (0.3%)	–	–	–	–
Total	17 (5.5%)	61 (20%)	2 (0.6%)	11 (3.6%)	1 (0.3%)	2 (0.6%)

*CD* Clavien-Dindo classification, *DVT* deep venous thrombosis, *TCI*, transient ischemic attack

6% of patients who underwent MW experienced a major (CD ≥ III) complication compared to 5% following RAPN. The most common post operative complication after MW treatment was postoperative infection and pain, while it was infection and bleeding following RAPN, Table 2. In uni- and multivariable logistic regression analyses, there was no significant difference in the risk of experiencing any or a major postoperative complication between patients undergoing MW or RAPN, Table 3. The same univariable and multivariable logistic regression analyses were also performed on the subset of patients included in the oncological analyses (51 treated with MW and 266 with RAPN), which also showed no significant difference in the risk of any or a major postoperative complication.

**Table 3:** Logistic regression analyses for the risk of a complication

Characteristic	All complications			CD ≥ 3		
	OR	95% CI	*p* value	OR	95% CI	*p* value
*Univariate analysis*						
*Treatment*						
PN	–	–		–	–	
MW	0.95	0.52, 1.67	0.9	1.14	0.32, 3.23	0.8
*Age*						
<75	–	–		–	–	
≥75	1.15	0.65, 2.01	0.6	1.16	0.32, 3.30	0.8
*Gender*						
Female	–	–		–	–	
Male	1.03	0.64, 1.68	0.9	4.13	1.17, 26.3	0.060
*CCI*						
0	–	–		–	–	
1	0.69	0.39, 1.17	0.2	0.53	0.12, 1.76	0.3
≥2	1.44	0.83, 2.49	0.2	1.82	0.64, 4.93	0.2
*ASA*						
2	–	–		–	–	
≥3	1.14	0.72, 1.79	0.6	1.87	0.75, 4.68	0.2
*BMI*						
Normal	–	–		–	–	
Pre-obesity	0.66	0.38, 1.15	0.14	0.66	0.21, 2.11	0.5
Obese	0.64	0.35, 1.15	0.14	0.73	0.22, 2.40	0.6
*Kidney function*						
CKD 1	–	–		–	–	
CKD 2	0.53	0.33, 0.85	0.009	0.99	0.35, 2.90	> 0.9
CKD ≥ 3	0.79	0.39, 1.56	0.5	2.48	0.70, 8.18	0.14
*Smoking*						
Never	–	–		–	–	
Smoker	1.36	0.76, 2.44	0.3	2.60	0.76, 10.2	0.14
Former smoker	1.60	0.95, 2.69	0.077	2.20	0.70, 8.28	0.2
*Tumor size (mm)*						
0-19.9	–	–		–	–	
20-29.9	1.83	0.96, 3.65	0.074	1.26	0.35, 5.88	0.7
30–40	1.68	0.88, 3.34	0.13	1.37	0.39, 6.31	0.6
*Multivariate analysis*						
*Treatment*						
PN	–	–				
MW	0.73	0.39, 1.33	0.3			
*CCI*						
0	–	–				
1	0.71	0.41, 1.21	0.2			
≥2	1.49	0.84, 2.62	0.2			

*CD* Clavien–Dindo classification score, *HR* hazard ratio, *CI* confidence interval, *PN* robot-assisted laparoscopic partial nephrectomy, *MW* microwave ablation, *ASA* American Society of Anaesthesiology score, *CKD* chronic kidney disease stage

Patients who underwent MW had a significantly smaller loss in eGFR one year after treatment compared to patients who underwent RAPN (MW: 2 mL/min/1.73 m² vs. RAPN: 3 mL/min/1.73 m², *p* = 0.03).

**Fig. 1:**
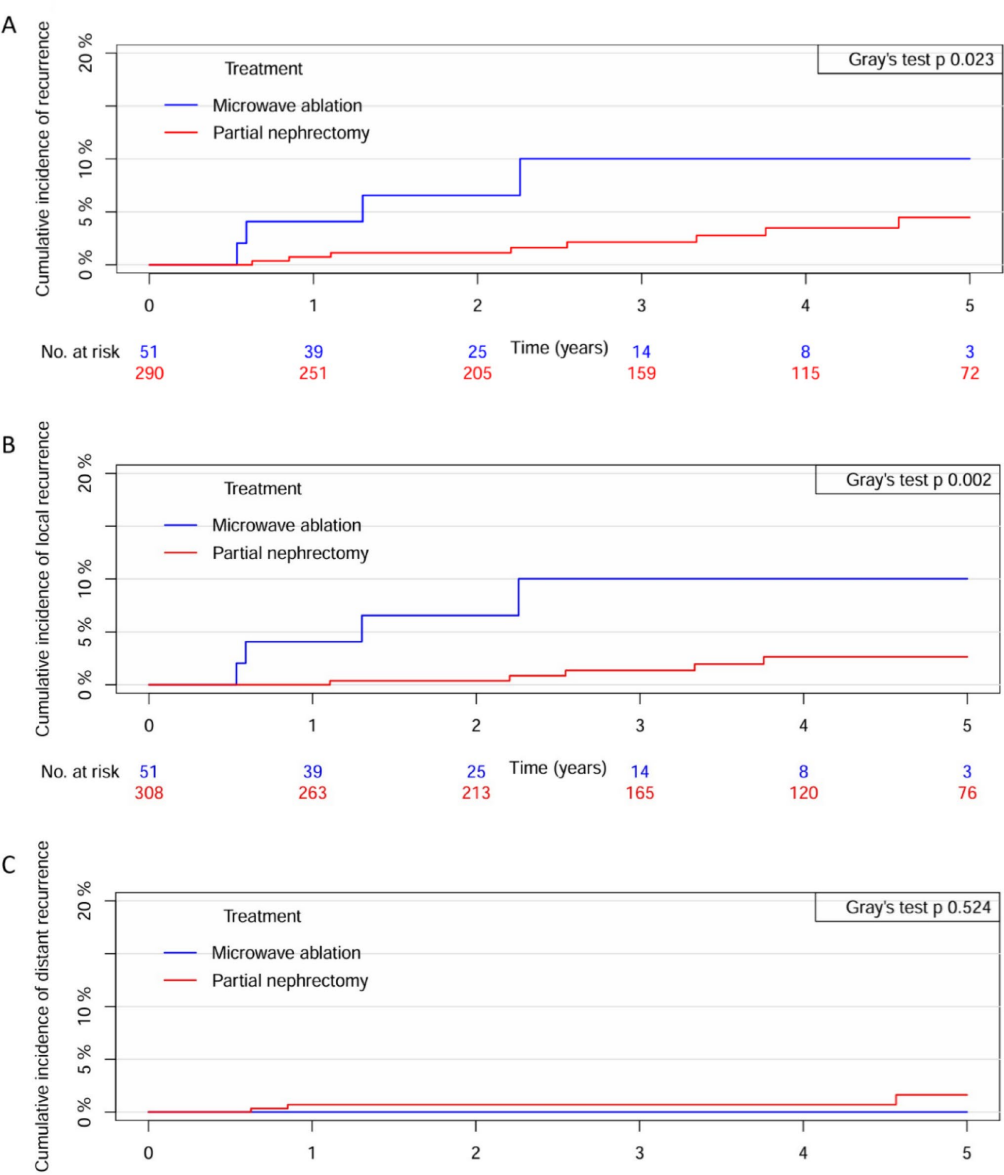
Cumulative incidence of **A** any recurrence, **B** local recurrence, and **C** distant recurrence following microwave ablation versus robot-assisted partial nephrectomy

In the group of patients treated with MW there was 4 (8%) recurrences in total, all of which were in the ablation area, i.e. local. Two patients were treated with re-MW ablation, one patient underwent nephrectomy and one underwent surveillance. There were 12 (4%) recurrences in total for patients treated with RAPN, of which 7 were local and 5 distant. The local recurrences were treated with either MW (3 patients) or nephrectomy (4 patients). The distant recurrences were managed with resection, stereotactic body radiation therapy, immunotherapy or surveillance. The 5-year cumulative incidence of any recurrence was 10% for MW versus 4% for RAPN, Fig. 1A.

The 5-year risk of local recurrence was 10% for MW versus 3% for RAPN, Fig. 1B. However, there was no difference in the 5-year incidence of a distant recurrence, with an incidence of 0% for MW and 2% for RAPN, respectively, Fig. 1C. In univariate Cox regression analyses, patients treated with MW had a higher risk of experiencing any recurrence and a local recurrence compared to those who underwent RAPN, HR 4.24 (95% CI 1.26, 14.3) and HR 6.95 (95% CI 1.83, 26.4), respectively. There was no difference in the risk of a distant recurrence between treatment strategies. There were no cancer specific deaths in either of the groups.

## Discussion

In this retrospective study comparing CT-guided MW with RAPN for T1a RCC, we found that patients who underwent MW spent fewer days in the hospital and were typically discharged the same day or the day after the intervention (1 to 2 days in hospital) compared to patients who underwent RAPN, who typically spent 2 to 4 days in hospital. The risk of any or a CD ≥ III postoperative complication was similar for both treatment modalities. A minor reduction in eGFR was observed in patients treated with MW and RAPN one year postoperatively with no clinically significant difference between the two treatment modalities. Treatment with MW resulted in a significantly higher risk of a local recurrence compared to RAPN, but this did not transcend into an increased risk of distant metastasis.

The main limitations of this study are the retrospective design and the lack of a protocol for treatment selection. Thus, there were significant differences between the two treatment groups, with MW patients being older, having more comorbidities, and possessing a lower baseline renal function. We tried to account for this difference by excluding patients with ASA 1 and performing multivariable analysis. Still there are additional biases which we were unable to control for due to the number of events, especially for oncological analyses where there were too few events for any meaningful multivariable analyses. The oncological outcomes might also be biased by the difference in median follow-up time, as non-random censoring and reduced statistical power may be more pronounced. Another limitation of the study is that the study may be underpowered. To detect a significant difference in recurrence rates between RAPN (4%) and MW (8%), with 80% power and a two-sided significance level of 5%, a total of 553 patients per arm would be required. Similarly, to identify a significant difference in major complications—assuming a rate of 5% in the RAPN group and 6% in the MW group—8158 patients per arm would be necessary under the same statistical assumptions. These calculations clearly illustrate that the current study is underpowered to detect such differences. However, they also underscore that the absolute differences in major complication rates between the two modalities are minimal, even though patients undergoing MW were generally older and had a higher comorbidity burden. Given these limitations, should the findings be interpreted with caution.

The main strength of this study is the complete follow-up. Another strength is that the cohorts were recruited from the same background population and treated at the same hospital, thus receiving the postoperative care at the same ward.

Our results are in line with the few previous publications who have compared CT-guided MW to RAPN. Lucignani et al. compared 62 patients who underwent CT-guided MW to 109 patients treated with RAPN and found patients undergoing MW had a shorter post operative hospital stay and no difference in the rate of CD ≥ III complications [13]. They also found that there were more recurrences, but this did not result in a poorer cancer-specific survival for patients undergoing MW [13]. Chlorogiannis et al. found no difference in recurrence-free survival and distant metastasis-free survival in a propensity score-matched study comparing 71 patients who underwent CT-guided MW to RAPN, with a median follow-up of 80 (MW) and 55 (RAPN) months, respectively [14]. Other studies comparing MW to PN, have also included laparoscopic and open approaches for PN, making it difficult to compare the safety profile of MW to PN. None of these studies showed a difference in overall survival, but some showed an increase in the risk of local recurrences [12, 21, 22].

RCC is a heterogenous disease and therefore requires a tailored treatment strategy. Some small tumors are indolent, thus curative treatment of small RCC carries a risk of overtreatment [23–26]. Active surveillance can mitigate this risk of overtreatment [6, 7]. However, not all tumors are suitable for active surveillance, either because of the nature of the tumor or because of patient preference, with some patients preferring the better chance of cure associated with treatment of smaller stage and grade RCCs and accept the inherent risks of active treatment [27, 28]. PN is the preferred curative treatment modality of T1a RCC, however, comparative studies between different treatment modalities are needed. The current study sheds light on safety profiles and oncological outcomes following two different curative treatment alternatives for T1a RCC. While the design does not allow for a clear-cut answer to which method to use, it helps to shed light on potential benefits and disadvantages that could help guide treatment selection. There was a similar risk of postintervention complication although patients who underwent MW where older, had more comorbidity and thus likely frailer. We can only speculate if these patients would have had similar safety outcomes had they undergone RAPN. The fact that we did not see an increased risk of distant recurrences further justify the consideration of MW as a viable option when RAPN is not possible or not advisable. But we cannot consider MW to be considered an equal alternative to RAPN, because of the higher risk of local recurrence following MW. However, the increased risk of local recurrences could be partly mitigated by the high success rate of repeat ablation [29]. Currently, it can only be speculated if the increased risk of local recurrence could result in worse oncological outcomes, which first manifests with even longer follow-up. However, the potential benefit of shorter hospital stay, may indicate that the recuperation is easier following MW and potentially more cost-effective than RAPN [30]. While the reduced fall in eGFR for MW patients was too small to be clinically significant, it suggests a gentler approach that spares more nephrons. Taken together, this may indicate that MW is a more compelling treatment option for frailer patients or patients where you are worried about their ability to recuperate physically and mentally after surgery. The implementation of visual aids for RAPN might mitigate these differences by enabling a more precise resection [31–33]. Future studies investigating quality of life directly comparing ablation with RAPN, could further enlighten patient’s perspective of the recuperation period.

## Conclusion

In this retrospective study we found that patients who underwent CT-guided MW for T1a RCC had a similar risk of experiencing a postintervention complication, an increased risk of having a local recurrence but no difference in the risk of distant recurrences compared to patients who underwent RAPN. Although there are major differences between the groups, these findings indicate that CT-guided MW is a feasible treatment option for patients with T1a RCC who are poor surgical candidates.

## Data Availability

No datasets were generated or analysed during the current study.
